# Effects of a life-skills-based prevention program on self-esteem and risk behaviors in adolescents: a pilot study

**DOI:** 10.1186/s40359-019-0358-0

**Published:** 2019-12-17

**Authors:** Virginie Moulier, Hélène Guinet, Zorica Kovacevic, Zohra Bel-Abbass, Yacine Benamara, Nadhirati Zile, Arezki Ourrad, Pilar Arcella-Giraux, Emmanuel Meunier, Fanny Thomas, Dominique Januel

**Affiliations:** 1 Unité de Recherche Clinique, EPS Ville Evrard, Neuilly-sur-Marne, France; 2 Service hospitalo-universitaire de psychiatrie adulte, CH du Rouvray, Sotteville-lès-Rouen, France; 3 APCIS, Stains, France; 4 Agence Régionale de Santé (ARS), Délégation Départementale de Seine-Saint-Denis, Bobigny, France; 5 Mission Métropolitaine de Prévention des conduites à risque, Pantin, France

**Keywords:** Prevention, Life-skills, Risk behaviors, Self-esteem, Adolescent

## Abstract

**Background:**

Risk behaviors among young people are a major social and public health issue. This study aims to assess the impact of a life-skills-based prevention program (called *Mission Papillagou*) on self-esteem, well-being, and risk behaviors among adolescents.

**Method:**

In a two-arm controlled study involving 520 school pupils aged between 10 and 15 years old, participants taking part in the prevention program (the Papillagou group) were compared to pupils who did not take part (the control group). Two assessment sessions were performed, one at baseline, and one after either the *Mission Papillagou* program (Papillagou group) or usual lessons (control group). Participants self-reported on their self-esteem, well-being, behaviors, interests and opinions.

**Results:**

The *Mission Papillagou* program significantly improved Self-Esteem scores (ηρ^2^ = .035). Well-being (Cramér’s V = .14) and mood (“feeling of depression”: Cramér’s V = .503; “feeling hopelessness about the future”: Cramér’s V = .357; “waking up at night”: Cramér’s V = .343) also improved in the Papillagou group compared to the control group. Regarding risk behaviors, the prevention program produced a decrease in the frequency of insults (Cramér’s V = .267) and rumor-spreading (Cramér’s V = .440), and a change of opinion toward the possibility of smoking an electronic cigarette in the future (Cramér’s V = .372).

**Conclusion:**

This study suggests that life-skills-based risk prevention programs are effective.

## Background

Adolescence is a crucial period of human development, characterized by psychological, biological and behavioral changes, the establishment of self-identity, and an increase in risk behaviors. These risk behaviors are defined as ways of acting that are seen as potentially damaging to the health, such as violence, school bullying, and consuming toxic substances (tobacco, cannabis, alcohol, etc.). A 2013/2014 collaborative international survey by the World Health Organization reported epidemiological data about risk behaviors in middle-schoolers, aged from 11 to 15, in 42 countries across Europe and North America [1]. Its findings stated that risk behaviors occurred from the beginning of adolescence (11 years old) and their prevalence could increase with age. In France, the number of adolescents who smoke tobacco at least once a week increased from 1% (for boys and girls) at 11 years old to 18% (for boys) and 20% (for girls) at 15 years old. Regarding alcohol use, 1% of female and 4% of male 11-year-old adolescents reported drinking alcohol at least once a week. This figure reached 8% among female and 16% among male 15-year-olds. Moreover, 6% of 15-year-old females and 7% of 15-year-old males reported their first experience of being drunk at age 13 or younger. With regards to cannabis use, France is at the top of the list of countries: 26% of female and 29% of male 15-year-olds reported to have consumed cannabis [1]. Regarding interpersonal risk behaviors, 7% of female and 13% of male 13-year-olds indicated having bullied other children (compared with 6% of female and 8% of male 11-year-olds), and 9% of female and 11% of male 13-year-olds reported having been bullied. However, physical confrontations tended to become less frequent from 11 to 13 years old: 20 to 12% for males and 8 to 6% for females. Finally, this survey highlighted that risk behaviors are major social and public health problems among young people. Indeed, risk behaviors induce adverse physical and mental health consequences in later adolescence or adulthood, including poor general health, addiction, anxiety, depression, and suicidal ideation [2–5], as well as the possibility of brain damage [6].

The development of risk behaviors from early adolescence could be explained by an increase in sensation-seeking during puberty, due to a remodeling of the brain’s dopaminergic system, which is involved in reward and motivation processing [7]. This sensation-seeking may be modulated by various factors, which either exacerbate or protect against risk behaviors. A psychosocial framework has been suggested to understand the development of risk behavior in adolescence [8]. This study broke down risk and protective factors into five domains: biological/genetics, social environment, perceived environment, personality, and behavior. According to this framework, low self-esteem, poverty, or poor school work, are among the risk factors promoting risk behaviors. Conversely, having a cohesive family, placing a high value on health and/or involvement in school, are protective factors.

Appropriate primary prevention programs may help prevent the initiation and development of risk behaviors from early adolescence. According to a report on the prevention of risk behaviors at schools by the *Institut National de Prévention et d’Education de la Santé* (INPES, France) [9], the most effective interventions prioritize the active and interactive participation of pupils, either through role-play or practical work on emotions. These methods are not limited to the mere transmission of information, and are based on the development and strengthening of life skills. Life skills are defined as abilities that enable individuals to deal effectively with the demands and challenges of everyday life, such as problem solving, critical thinking, empathy, interpersonal skills, and coping with both emotions and stress [10]. Their acquisition promotes positive mental well-being, better relationships, and healthier behaviors. It also contributes to developing protective factors against risk behaviors, such as self-esteem. Self-esteem is defined as the overall appraisal that a person makes of his/her own worth, and represents a critical component of mental health [11]. Low self-esteem is related to risk behaviors such as delinquency and antisocial behaviors in 11- and 13-year-old adolescents [12]. Moreover, 11-year-olds with lower self-esteem are more likely to display aggressive behavior at the age of 13. Low self-esteem also predicts the onset of smoking [13] and toxic substance consumption, including marijuana, crack, or cocaine [14].

Life-skills-based interventions must be adapted, as closely as possible, to their target populations. The most effective intervention programs for children are those that take place in the school environment, as early as possible in the development of risk behaviors [15]. In addition, Velasco et al. highlighted the importance of implementing prevention programs in middle schools before adolescents begin experimenting with drugs [16]. However, in France, finding time during the school curriculum to arrange these prevention programs is often a challenge. Some effective prevention programs are particularly time-consuming, as they consist of 9 to 15 sessions per year [16].

Since 2012, a life-skills-based program called *Mission Papillagou* has been implemented in several schools that are based in economically disadvantaged Parisian suburbs. *Mission Papillagou* aims to reinforce young teenagers’ self-esteem, to improve the atmosphere in the classroom, to prevent risk behaviors, and to develop interpersonal skills. This program promotes abilities such as: (i) solving problems and making decisions; (ii) communicating effectively and being socially comfortable; (iii) thinking creatively and critically; (iv) empathizing, and becoming aware of one’s emotions; and (v) coping with stress and being self-aware. Improving life skills could raise self-esteem among young people, and could constitute a protective factor against risk behaviors.

Based on a science-fiction story, *Mission Papillagou* consists of a set of group activities which are performed over three separate three-hour sessions (9 h in total) over 2 weeks. The different activities, their duration and the life skills that are promoted during each session are described in Table 1. The first session focuses on harmful behaviors (influence, manipulation, spreading rumors), the second session on the importance of cooperating within a group, and the third session on confidence and expressing feelings. The program is administered by six facilitators (one educator-supervisor and five nursing students) who have been trained in the delivery of the program. Each session is divided into two steps. The first step is a role-play game with a series of puzzles to solve in small groups (five or six pupils), each group being supervised by a nursing student. The second step, led by the educator, consists of debates based on the topics covered during the first step’s activities. Promoting autonomy and empowerment, *Mission Papillagou* encourages children to experience a series of situations or to solve problems themselves. The facilitators help children to develop their own preventive measures.

**Table 1:** Characteristics and components of Mission Papillagou program

	Day 1			Day 2			Day 3		
	In small groups		With all pupils of class	In small groups		With all pupils of class	In small groups		With all pupils of class
	Activity 1	Activity 2		Activity 1	Activity 2		Activity 1	Activity 2	
Duration	45 min	45 min	55 min	50 min	50 min	55 min	50 min	50 min	55 min
Content	Rumor	Social pressure	Debate: how to identify and prevent behaviors that affect social life; how to control impulsivity	Gender	Coping with frustration and anger	Debate: how to develop trusting relationships and manage emotions	Encouraging others	Benefits of being a child, benefits of being an adult	Debate: how to support each other, avoid risk behaviors, and deal with adolescence
Life skills	Critical thinking and empathy	Coping with stress and emotion	Critical thinking, empathy, coping with stress and emotion	Critical thinking and empathy	Relationships, coping with stress and emotion	Empathy, relationships, coping with stress and emotion	Relationships and self-awareness	Self-awareness	Empathy, relationships, and self-awareness

Notes. Each session started with an introduction of 25 min (Day 1), or 15 min (Day 2 and Day 3)

Since 2012, 95 classes, i.e. 2355 pupils, in the Seine-Saint-Denis district (an economically challenged suburb, north-east of Paris) have taken part in this program. *Mission Papillagou* therefore needed to be evaluated to highlight its impact on risk behaviors in adolescents. The aim of this study was to examine the effectiveness of *Mission Papillagou* on the self-esteem, well-being and risk behaviors of young adolescents, compared to a control group who did not take part in the program.

## Methods

### Participants

**Fig. 1:**
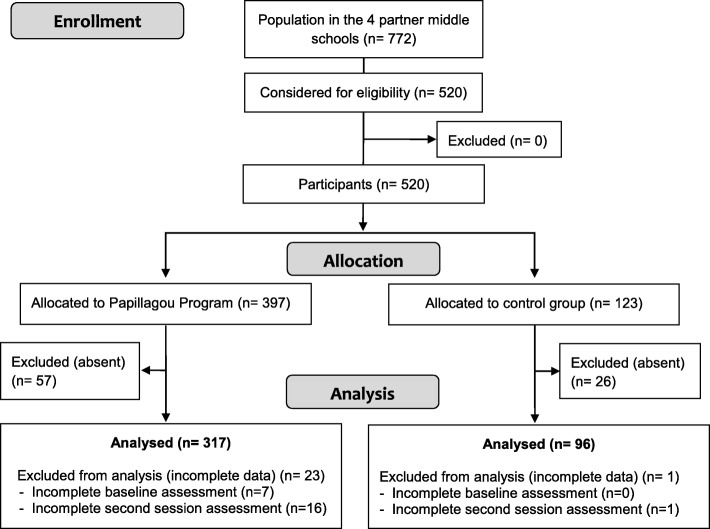
Flow diagram

The inclusion criteria were: (i) being pupils from sixth- or seventh-grade classes from four middle schools in two economically disadvantaged neighborhoods of Seine-Saint-Denis (a suburb of Paris) who volunteered to participate in the *Mission Papillagou* program, (ii) being between 10 and 15 years old, and (iii) reading and writing French well enough to complete the questionnaires. As shown in Fig. 1, 520 pupils were considered eligible for the study, in which a total of 22 sixth- and seventh-grade classes took part. In total, 22 sixth- and seventh-grade classes were assessed. All children agreed to participate in the program and assessments. The program consisted of some role plays, and it took place during school hours. The children’s parents were all in favor of having their children participate in a program that could help reduce risky behaviors.

The research protocol was approved by each school principal. Parents and children received clear, accurate and detailed information about the protocol, and gave written consent to participate. Data confidentiality was guaranteed by an identification number. For ethical reasons, the *Mission Papillagou* program was offered to the control group following the end of the study (the program occured in the months following the assessment sessions or the year after).

### Study design

A two-arm controlled trial was conducted. Nine sixth-grade classes and eight seventh-grade classes from three middle schools made up the Papillagou group (who received the prevention program); three sixth-grade classes and two seventh-grade classes from the other middle school made up the control group (who did not receive the program). It was necessary that the control group came from a different (albeit socio-economically similar) middle school, to prevent the effects of *Mission Papillagou* from spreading to other classes through time shared outside of the classroom by the pupils.

### Procedure

Two assessment sessions were performed: one session at baseline (both groups) and one session after either the Papillagou program (Papillagou group) or lessons as usual (control group). The second session took place between 2 weeks and 1 month after the end of the program (depending on school holidays). On average, 43 (±21) days separated the two assessments. The evaluations consisted of self-questionnaires and did not require the help of adults to be filled in. To avoid bias, the questionnaires were handed out to participants by someone other than the educator who administered the program when possible. This was a member of APCIS (*Accueils Préventions Cultures: Intercommunautaire et Solidaire,* *apcis@wanadoo.fr*, an association involved with specially adapting the *Mission Papillagou* program for middle schools). Data was captured on a computer by two members of a research team (from *Unité de Recherche Clinique de Ville-Evrard*) who also did not administer the program.

### Prevention program

The *Mission Papillagou* prevention program was specifically adapted for middle schoolers by APCIS and MMPCR (*Mission Métropolitaine de Prévention des Conduites à Risque*) from a program called *Mission Papillagou and Croc’Lune’s Children*, which was created in 1997 by the National Association for the Prevention of Alcoholism and Addiction in collaboration with The Swiss Institute for the Prevention of Alcohol and Drug Problems [17]. The adapted program was administered by an APCIS educator, along with five nursing students. Prior to implementing the program, the MMPCR organized a six-day training course for the nursing students, which included: i) a presentation of the *Mission Papillagou* program (half a day); ii) a description of its content and practical application through role-play games (five half days); iii) focusing on the problem of violence in schools, including how to prevent it (two half days); iv) focusing on addictive behaviors and their prevention (half a day); v) development of life skills (two half days). Each session ended with a debriefing.

In addition to this training course, when the *Mission Papillagou* program was carried out in schools, an APCIS educator was on hand to help the nursing students implement the program correctly.

### Assessments

At baseline, and after the two-week *Mission Papillagou* program/lessons-as-usual, self-esteem, well-being, behaviors, interests and pupils’ opinions were assessed through self-reporting. The assessments took place at school, and lasted for around 1 h each time.

#### Primary outcome measure: self-esteem assessment

Self-esteem was measured with the Self-Esteem Scale of Toulouse (ETES), a validated self-administered questionnaire of 60 items [18]. Participants were asked to rate their agreement using a five-point Likert scale (from “totally agree” to “totally disagree”). Five sub-categories were distinguished: Emotional Self, Social Self, Scholarly Self, Physical Self, and Future Self. The Emotional Self score represented the participants’ control over their emotions and impulses. The Social Self score represented interactions with others (family, friends, etc.), and the feeling of being recognized socially. The Scholarly Self score represented attitudes, behavior and school performance. The Physical Self score referred to how each individual believed their physical appearance was viewed by others, how they viewed their own physical appearance, their own sports skills, and their own attractiveness. The Future Self score referred to how each child saw themselves in adulthood. The sum of these five scores constituted the total ETES score. Regarding its psychometric characteristics, this scale exhibited a good internal consistency in the whole sample (α = 0.81) and in the two samples (Papillagou group: α = 0.80; control group: α = 0.84).

#### Secondary outcome measures

##### Well-being assessment

An unvalidated Visual Analog Scale (VAS) was used to measure the adolescents’ sense of well-being. The VAS comprised of a horizontal line, 10 cm in length, anchored by two well-being verbal descriptors, from “I feel awful” on the left, to “I feel very well” on the right. The children were instructed to draw a cross on the line.

##### Health and risk behavior assessment

A self-administrated questionnaire called “PEPS”, which had been adapted from a French national survey by Choquet & Ledoux [19] and a study by Choquet & Lagadic [20], was used to investigate health and risk behaviors. This questionnaire consisted of assertions, divided into six sections: i) social and educational situation; ii) relationships; iii) mental and physical health (for example, *“in the last two weeks, I have felt depressed”* or *“in the last two weeks, I have had headaches”*); iv) risk behaviors, including toxic substance consumption (for example, *“have you ever smoked cigarettes? If so, how many cigarettes a day/ a week/ a month?”*), anti-social behaviors such as absenteeism (for example, “*in the last two weeks, I have skipped school*”), physical violence (for example, *“in the last two weeks, I have been in a fight”)*, and verbal violence (for example, *“in the last two weeks, I have spread a rumor”* or *“in the last 2 weeks, I have insulted someone in school*); v) activities and interests; and vi) opinions. Subjects had to rate the frequency of each assertion.

### Sample size

Since the threshold of discrimination for changes in health-related instruments appears to be approximately one half a Standard Deviation (SD) [21], the sample size needed to detect a meaningful difference on the Self-Esteem Scale of Toulouse (ETES) between the two groups was 92 subjects per group, with a 90% power (alpha = 0.05, two-tail). Considering the high rate of absenteeism in these schools (20 to 25%), we included at least 123 subjects per group. Given the direct benefits of the Mission Papillagou program for children, the number of subjects included in the Papillagou group was greater than the minimum number of participants required.

### Statistical analyses

Statistical analyses were performed with SPSS®25 Software (Chicago, IL). Data entry on a computer was done by two people (one reading the data aloud and the other inputting it). Then, the accuracy of the data entry was randomly checked. Only data from pupils who completed the program was included in the analyses (per-protocol analysis). A mixed analysis of variance (ANOVA) was performed to analyze Total Self-Esteem score, and a multivariate analysis of variance (MANOVA) with bootstrapping was performed to analyze the dimensions of Self-Esteem (emotional, social, scholarly, physical and future self). Well-being was transformed into a categorical variable and was analyzed with a chi-squared test. Regarding the PEPS questionnaire, the categorical variables were recorded as binary (yes/no) variables in terms of improvement at the second assessment compared to the first. The proportion of subjects who improved was compared between the two groups using the chi-squared test. If the criteria for using the chi-squared test were not met, Fisher’s exact test was used.

Effect sizes were measured through partial eta squared (ηρ^2^) for mixed ANOVA and MANOVA (0.0099 (small); 0.0588 (medium); 0.1379 (large)) and Cramér’s V for chi-squared or Fisher’s exact tests (for one degree of freedom: 0.1 (small); 0.3 (medium); 0.5 (large)). Benchmarks to define small, medium and large have been suggested by Cohen [22].

## Results

### Participant characteristics

The data from 413 pupils who participated in all assessments was analyzed: 317 in the Papillagou group (who took part in the *Mission Papillagou* program), and 96 in the control group (who did not take part in the program). The participants were aged between 10 and 15 years old: mean (Standard Deviation, SD) age = 11.82 (.86) in the Papillagou group; mean (SD) age = 11.83 (.88) in the control group (no significant difference between groups: t_410_ = 1.011; *p* = .313). In terms of gender distribution, there were 179 males/138 females in the Papillagou group and 47 males/49 females in the control group (no significant difference between groups: X^2^(1) = 1.417; *p* = .234). Regarding toxic substance consumption in the whole sample at the baseline, 16.5% of the pupils had already drunk alcohol (boy-girl ratio = 58/42%), 11.1% had already tried smoking tobacco (boy-girl ratio = 67/33%), 8.5% had already tried electronic cigarettes (boy-girl ratio = 71/29%) and 3.4% had already tried cannabis (boy-girl ratio = 69/31%). Among the adolescents who had already consumed toxic substances, 2.7% smoked cigarettes at least once a week (*n* = 11), 8.2% smoked hookah at least once a week (*n* = 33), 4.5% smoked electronic cigarettes at least once a week (*n* = 18), and 0.5% smoked cannabis at least once a week (*n* = 2). 2.5% of the pupils also reported regularly drinking alcohol (*n* = 10). Regarding interpersonal risk behaviors, 19.8% reported having skipped school at least once in the previous two weeks (*n* = 79), 44.7% had insulted someone in school (*n* = 178) and 18.4% reported fighting at least once in the previous two weeks (*n* = 73).

### Effect of the program on self-esteem

No significant difference was found between the two groups at baseline for all the variables of self-esteem (F[5407] = .510; *p* = .769). The Pearson correlation was used to study the relationship between self-esteem and age. Self-esteem was negatively correlated with the age of the adolescents (*r* = −.117; *p* = .017).

Regarding total Self-Esteem score, the mixed ANOVA revealed a significant group-by-time interaction effect (F[1411] = 8.89; *p* = .003; ηρ^2^ = .021). Significant main effect of time (F[1411] = 6.31; *p* = .012; ηρ^2^ = .015) and main effect of group (F[1411] = 5.59; *p* = .019; ηρ^2^ = .013) were found. There was no significant difference between the two groups at baseline (m(SD)_Papillagou_ = 218.32(23.21) and m(SD)_Control_ = 215.28(26.17); t_411_ = 1.091; *p* = .276), but there was a significant difference after the Program (m(SD)_Papillagou_ = 218.85(25.13) and m(SD)_Control_ = 209.18(28.56); t_411_ = 3.197; *p* = .001).

Regarding different Self-Esteem dimensions, MANOVA revealed that the program had a significant overall effect on Self-Esteem scores (F[5407] = 2.938; *p* = .013; ηρ^2^ = .035), especially on the Physical Self score. Table 2 includes the means (SD) of Self-Esteem scores, as well as the MANOVA results for each Self-Esteem score (Wilks’Lambda test).

**Table 2:** Effect of the program on self-esteem

Self-esteem scores	*First Session*		*Second Session*				
	Papillagou group	Control group	Papillagou group	Control group	Wilks’Lambda	*p*-value	Effect size
	Mean *(SD)*	Mean *(SD)*	Mean *(SD)*	Mean *(SD)*	F(1,411)		ηρ^2^
Emotional self	44.43 (6.95)	43.46 (7.30)	44.46 (6.94)	42.32 (7.76)	2.73	*p* = .099	*.007*
Social self	45.01 (5.83)	44.92 (5.52)	44.34 (6.08)	43.44 (6.91)	1.26	*p* = .263	.003
Scholarly self	41.83 (8.65)	40.63 (9.27)	42.29 (8.66)	39.69 (8.56)	3.54	*p = .061*	.009
Physical self	44.61 (7.80)	44.36 (9.39)	45.41 (7.89)	42.44 (8.89)	14.11	***p*** < .001***	.033
Future self	42.44 (4.84)	41.92 (6.28)	42.35 (5.07)	41.29 (6.30)	0.73	*p* = .393	.002

Legends. Means and Standard deviations are reported for each group. Bolded values indicate *p* ≤ .05

* *p* ≤ .05; ** *p* ≤ .01; *** *p* ≤ .001

Similar results were observed when age was introduced as a covariate in the analysis.

### Effect of the program on well-being

No significant difference in well-being was found between the two groups at baseline: mean (SD) _Papillagou_ = 8.1 (2.5) and mean (SD) _Control_ = 8.2 (2.6) (t_411_ = .453; *p* = .651).

Between both assessment sessions, well-being improved in 44% of Papillagou group participants (versus 32% in the control group), remained stable in 19% (versus 15% in the control group) and decreased in 37% (versus 53% in the control group). There was a significant change in well-being between the two groups when using the chi-squared test (X^2^ (2) = 8.048; *p* = .018; Cramér’s V = .14).

### Effect of the program on mood

The percentage of adolescents in each group reporting symptoms related to mood at baseline is reported in Table 3. No significant difference was found between the two groups at baseline when using the chi-squared test. Among pupils reporting mood symptoms at baseline, either the chi-squared test or Fisher’s exact test was used to compare improvements in the two groups at the second assessment session. After the *Mission Papillagou* program, adolescents showed a significant improvement in comparison with the control group in: i) waking up at night (X^2^ (2) = 10.679; *p* = .001; Cramér’s V = .343), ii) feelings of depression (*p* = .019; Cramér’s V = .503), iii) feeling hopelessness about the future (*p* = .035; Cramér’s V = .357), and iv) a non-significant tendency for feelings of sadness (*p* = .056; Cramér’s V = .426; Table 3).

**Table 3:** Frequency and course of mood symptoms

*In the last 2 weeks*	Papillagou group (*n* = 317)		Control group (*n* = 96)				
	Frequency at the first assessment ± 95% CI	Percentage of children who reported feeling better at the second assessment	Frequency at the first assessment ± 95% CI	Percentage of children who reported feeling better at the second assessment	Statistical value	*p*-value	Effect size Cramér’s V
Having trouble falling asleep at night	24.61% ± 4.74 (*n* = 78)	51.28%	27.08% ± 8.89 (*n* = 26)	42.31%	X2 (1) = .628	.428	.078
Waking up at night	21.14% ± 4.49 (*n* = 67)	59.70%	25% ± 8.66 (*n* = 24)	20.83%	X2 (1) = 10.679	.001***	.343
Reproaching themselves about something	14.20% ± 3.84 (*n* = 45)	68.89%	16.67% ± 7.46 (*n* = 16)	43.75%	X2 (1) = 3.176	.075	.228
Feeling lethargic	14.20% ± 3.84 (*n* = 45)	53.33%	8.33% ± 5.53 (*n* = 8)	75.00%	Fisher exact	.441	.157
Being generally worried	10.09% ± 3.32 (*n* = 32)	53.13%	10.42% ± 6.11 (*n* = 10)	60.00%	Fisher exact	1.000	.059
Feeling depressed	7.26% ± 2.86 (*n* = 23)	69.57%	4.17% ± 3.98 (*n* = 4)	0.00%	Fisher exact	.019*	.503
Feeling hopelessness about the future	10.73% ± 3.41 (*n* = 34)	73.53%	7.29% ± 5.20 (*n* = 7)	28.57%	Fisher exact	.035*	.357
Feeling sad	7.57% ± 2.91 (*n* = 24)	66.67%	3.13% ± 3.48 (*n* = 3)	0.00%	Fisher exact	.056	.426

Legends. *CI* Confidence Interval. Bolded values indicate *p* < .05

* *p* ≤ .05; ** *p* ≤ .01; *** *p* ≤ .001

### Effect of the program on risk behaviors

The frequency of risk behaviors in each group at baseline is reported in Table 4. No significant difference was found between the two groups at baseline (chi-squared test) except for two items: i) spreading a rumor in school (X^2^(1) = 4.54; *p* = .033; and ii) stealing (X^2^(1) = 8.24; *p* = .004), with a higher frequency in the control group. Among pupils reporting risk behaviors at baseline, either the chi-squared test or Fisher’s exact test was used to compare the improvement between the two groups after the second assessment. After the *Mission Papillagou* program, adolescents showed a significant improvement in comparison with the control group in: i) spreading a rumor in school (X^2^ (1) = 10.656; *p* = .001; Cramér’s V = .440); ii) having been insulted in school  (X^2^ (1) = 8.147; *p* = .004; Cramér’s V = .267).

**Table 4:** Frequency and course of risk behaviors

*Occured at least once during the last 2 weeks*	Papillagou group (*n* = 317)		Control group (*n* = 96)				
	Frequency at the first assessment ± 95% CI	Percentage of children reporting a decrease at the second assessment	Frequency at the first assessment ± 95% CI	Percentage of children reporting a decrease at the second assessment	Statistical value	*p*-value	Effect size Cramér’s V
Skipping school	18.93% ± 4.31 (*n* = 60)	36.67%	21.88% ± 8.27 (*n* = 21)	23.81%	X2 (1) = 1.157	.282	.120
Arriving at school late	51.74% ± 5.50 (*n* = 164)	42.68%	58.33% ± 9.86 (*n* = 56)	35.71%	X2 (1) = .839	.360	.062
Insulting someone in school	42.27% ± 5.44 (*n* = 134)	35.07%	46.875% ± 9.98 (*n* = 45)	28.89%	X2 (1) = .578	.447	.057
Spreading a rumor around school	11.36% ± 3.49 (*n* = 36)	72.22%	19.79% ± 7.97 (*n* = 19)	26.32%	X2 (1) = 10.656	.001***	.440
Spreading a rumor on social networks	6.94% ± 2.80 (*n* = 22)	68.18%	6.25% ± 4.84 (*n* = 6)	33.33%	Fisher exact	.174	.293
Physical fighting	17.67% ± 4.20 (*n* = 56)	58.93%	19.79% ± 7.97 (*n* = 19)	42.11%	X2 (1) = 1.620	.203	.147
Stealing	6.31% ± 2.67 (*n* = 20)	45.00%	15.63% ± 7.26 (*n* = 15)	40.00%	X2 (1) = .088	.767	.050
Doing something illegal	6.62% ± 2.74 (*n* = 21)	57.14%	9.38% ± 5.83 (*n* = 9)	44.44%	Fisher exact	.694	.117
Bullying someone to obtain something	3.79% ± 2.10 (*n* = 12)	58.33%	6.25% ± 4.84 (*n* = 6)	66.67%	Fisher exact	1.000	.081
Have been insulted at school	26.50% ± 2.48 (*n* = 84)	53.6%	31.25% ± 9.27 (*n* = 30)	23.3%	X2 (1) = 8.147	.004**	.267
Have been physically assaulted at school	6.31% ± 2.68 (*n* = 20)	75.00%	7.29% ± 5.20 (*n* = 7)	42.86%	Fisher exact	.175	.299
Have been stolen from	7.89% ± 2.97 (*n* = 25)	76.00%	11.46% ± 6.37 (*n* = 11)	45.45%	Fisher exact	.124	.298
Have been bullied into giving away something	4.73% ± 2.34 (*n* = 15)	66.67%	7.29% ± 5.20 (*n* = 7)	28.57%	Fisher exact	.172	.356

Legends. *CI* Confidence Interval. Bolded values indicate *p* < .05

* *p* ≤ .05; ** *p* ≤ .01; *** *p* ≤ .001

Regarding the consumption of toxic substances, the number of substance users per group was insufficient to conduct statistical analyses. However, after the *Mission Papillagou* program, 57% of pupils no longer planned to smoke an electronic cigarette in the future, compared to 12% in the control group (Fisher Exact; *p* = .044; Cramér’s V = .372).

## Discussion

The purpose of this study was to evaluate the impact of the *Mission Papillagou* program on self-esteem, well-being and risk behaviors among middle-schoolers. The program was performed in the school environment over three separate three-hour sessions. It is designed to ameliorate and strengthen young people’s life skills, thus developing protective factors against risk behaviors. Our findings suggest an improvement in self-esteem, well-being, mood, and a reduction in some risk behaviors among adolescents who took part in the program, compared with the control group.

The *Mission Papillagou* program significantly improved both the total self-esteem and Physical Self scores. At the second assessment session, these mean self-esteem scores increased slightly in the Papillagou group, while they decreased in the control group. Self-esteem is highly associated with body image in young people [23–26]. Satisfaction with his/her own physical appearance can denote high self-esteem [23, 25, 26]. In this study, the improvement in total self-esteem among adolescents who participated to the program could therefore be due to a more positive attitude toward their own physical appearance. Although the topics covered during this program did not focus on physical appearance, role-play games allowed adolescents to act in front of their peers in order to develop self-acceptance and positive self-perception, while relativizing body changes related to puberty. Moreover, some of the activities covered during the program, such as stating classmates’ qualities and receiving compliments from them, can improve self-esteem and promote better relationships with others.

In addition, according to our results, self-esteem may be negatively correlated with age, but this result should be considered with caution because of its weak magnitude (*r* = −.117). Nevertheless this would tally with other studies that reported a decline in self-esteem [27], particularly among 12 and 13 year-olds [28, 29], due to the physical and psychological changes experienced during puberty. This suggests that the *Mission Papillagou* program could limit self-depreciation observed throughout adolescence. Other life-skills programs have reported a positive impact on self-esteem among young people [30–32]. For example, a French program called *ESPACE*, which focused on developing psychosocial skills and self-esteem in order to reduce the age of regular alcohol consumption among adolescents in middle schools, reported improved self-esteem (including self-confidence and body image) among adolescents who took part in the program compared to a control group [33]. This program consisted of 43 h of intervention over three years. However, the authors did not demonstrate a significant difference in alcohol consumption between both groups.

Low self-esteem has been shown to be significant in the etiology of psychiatric disorders such as depression and anxiety, as well as addictive disorders, particularly in adolescents and young adults [34]. Since self-esteem plays a major role in the adaptation of the individual to his or her environment, it is a protective factor against risks related to adolescent development, including toxic substance consumption [34]. Preserving self-esteem during early adolescence might have a long lasting effect, preventing the development of depressive symptoms in late adolescence and early adulthood. Indeed, a large prospective cohort study by Masselink et al. [35], which followed 2228 adolescents over several years, showed that low self-esteem was a vulnerability factor for developing depressive symptoms.

Together with boosting self-esteem, the *Mission Papillagou* program also significantly improved well-being and mood, in comparison with the control group. Mood is defined as a temporary state of mind, and is a component of well-being [36]. Self-esteem is both a protective factor and a strong predictor of mood and well-being [11, 37]. Life-skills based topics covered during the *Mission Papillagou* program, such as coping with stress and emotions, and developing better relationships with peers and adults, allowed adolescents to feel better about themselves and others. Thus, there were improvements in both their well-being and their mood (including better sleeping patterns, fewer depressive feelings, and more feelings of hope). Other prevention programs reported an improvement in well-being and lower levels of distress among program participants compared to a control group [16]. However, the ESPACE program, which aimed to promote self-esteem in adolescents, reported no difference in well-being, including current life satisfaction, feeling depressed, or feeling worried [33] .

Depression and feelings of unease in adolescents are of great cost to public health. In early adolescence, the prevalence of depression is around 2%, and it increases throughout adolescence to reach about 18% in early adulthood [38]. This program produced encouraging results to combat this, with a notably large effect size on depression. However our results need to be tested in future research using validated scales of mood and well-being.

The *Mission Papillagou* program also resulted in a decrease in the frequency of risk behaviors, specifically insults and the spread of malicious rumors. Verbal harassment and rumor spreading are part of bullying, which is a key contributor to global mental health issues [2]. Being a victim of bullying is especially associated with depression, reduced self-esteem, and anxiety, as well as a probable contributor to alcohol, tobacco and illicit drug use [2]. The effectiveness of the program on decreasing verbal harassment is therefore likely to improve mood, quality of life and self-esteem among potential victims of bullying. However, the program’s effect on the spread of malicious rumors should be treated with caution, because the pupils in the Papillagou group spread less rumors than those in the control group at the first assessment.

Regarding the program’s effect on toxic substance consumption, the sample of consumers was not large enough to perform statistical analyses. Nevertheless, the program induced a change of opinion toward the possibility of smoking an electronic cigarette in the future.

Our results are consistent with the *ESPACE* study, which reported a positive impact on self-esteem and psychosocial skills among pupils who took part in the program compared to a control group, but no difference regarding their consumption of toxic substances [33]. This latter finding can be explained by the early age (around 15 years old) of the participants, an age at which regular use of toxic substances affects only a limited number of young people.

Compared to other prevention programs, *Mission Papillagou* has the advantage of being shorter (thus it is easier to incorporate into the school curriculum), and less specialized (i.e. it does not focus on a single disorder). It addresses several issues by adapting to the specific problems encountered by the class. Finally, it is important to note that the *Mission Papillagou* program did not have any negative effects on participants.

However our outcomes should be treated with caution because the effect sizes were mostly small (self-esteem, well-being, frequency of insults) or medium (“feeling hopelessness about the future”, “waking up at night”, rumor-spreading and smoking an electronic cigarette in the future), except for the depressive feeling variable, which had a large effect size. This positive effect on mood may be explained by its lower inertia compared to more complex psychological concept, as self-esteem and well-being. The latter might require more time to show a larger fluctuation. The current study has some limitations. First, as mentioned above, there was a low proportion of toxic substance users among participants. This prevented any conclusion being drawn regarding the effect of the program on participants’ current toxic substance consumption. A study on a larger number of subjects would have made it possible. Secondly, regarding experimental design, schools were not randomly assigned in the Papillagou group or the control group for practical and organizational reasons, which could constitute a bias. Nevertheless, the schools that took part in this study had very similar socio-economic profiles. Moreover, participants and informants were not blind to study conditions. Thirdly, the scale used to assess well-being was not validated. A validated scale would have ensured greater reliability, and comparison with other studies. To our knowledge, there has never been a validated French-language scale to assess well-being in adolescents. Fourthly, days separating the two assessments were slightly different according to classrooms (depending on school holidays and availability schedule). In future studies, it would be better to control this factor more strictly.

Finally, the effect of the program was only assessed in the short term. It would be more valuable to assess the impact of the *Mission Papillagou* program over a longer period, such as 1 or 2 years. This project is currently under consideration, but it requires conducting a study with a larger cohort of children, because of high risk of lost to follow-up (move, change of school, school exclusion...).

## Conclusion

This study confirms the probable benefits of implementing risk prevention programs that promote life skills. Besides reducing risk behaviors, the *Mission Papillagou* program has a generally positive effect on young adolescents, especially on self-esteem, well-being and mood. The implementation of this type of program in schools should therefore be encouraged.

## Data Availability

The datasets used and analyzed during the current study are available from the corresponding author upon request.

## References

[1] World Health Organization (2014). Growing up unequal : gender and socioeconomic differences in young people’ s health and well-being.

[2] Moore SE, Norman RE, Suetani S (2017). Consequences of bullying victimization in childhood and adolescence: a systematic review and meta-analysis. World J Psychiatry.

[3] Due P, Holstein BE, Lynch J (2005). Bullying and symptoms among school-aged children: international comparative cross sectional study in 28 countries. Eur J Pub Health.

[4] Fontes MA, Bolla KI, Cunha PJ (2011). Cannabis use before age 15 and subsequent executive functioning. Br J Psychiatry.

[5] National Center for Chronic Disease Prevention and Health Promotion (US) Office on Smoking and Health (2012). Preventing tobacco use among youth and young adults. A report of the surgeon general.

[6] Ewing SWF, Sakhardande A, Blakemore S (2014). The effect of alcohol consumption on the adolescent brain: a systematic review of MRI and fMRI studies of alcohol-using youth. NeuroImage Clin.

[7] Steinberg L (2008). A social neuroscience perspective on adolescent risk-taking. Dev Rev.

[8] Jessor R (1991). Risk behavior in adolescence: a psychosocial framework for understanding and action. J Adolesc Health.

[9] Bantuelle M, Demeulemeester R (2008). Comportements à risque et santé : agir en milieu scolaire.

[10] World Health Organization. Life skills education for children and adolescents in schools. Introduction and guidelines to facilitate the developpement and implentation of life skills programmes. Geneva: World Health Organization; 1997.

[11] Mann M, Hosman CMH, Schaalma HP (2004). Self-esteem in a broad-spectrum approach for mental health promotion. Health Educ Res.

[12] Donnellan MB, Trzesniewski KH, Robins RW (2005). Low self-esteem is related to aggression, antisocial behavior, and delinquency. Psychol Sci.

[13] Carvajal SC, Wiatrek DE, Evans RI (2000). Psychosocial determinants of the onset and escalation of smoking: cross-sectional and prospective findings in multiethnic middle school samples. J Adolesc Health.

[14] Wheeler SB (2010). Effects of self-esteem and academic performance on adolescent decision-making: an examination of early sexual intercourse and illegal substance use. J Adolesc Health.

[15] Webster-Stratton C, Taylor T (2001). Nipping early risk factors in the bud: preventing substance abuse, delinquency, and violence in adolescence through interventions targeted at young children (0-8 years). Prev Sci.

[16] Velasco V, Griffin KW, Botvin GJ, Corrado Celata and Gruppo LST Lombardia (2017). Preventing adolescent substance use through an evidence-based program: effects of the Italian adaptation of life skills training. Prev Sci.

[17] Breton M, Catto S, Mordoj D, Menetrey A-C (1994). Papillagou and Croque Lune’s children. Institut Suisse de Prévention de l’Alcoolisme et Autres Toxicomanies.

[18] Oubrayrie N (1997). L’estime de soi de l’enfant et de l’adolescent. L’Echelle Toulousaine d’Estime de soi -E.T.E.S.- comme technique d’évaluation. Prat Psychol.

[19] Choquet M, Ledoux S (1994). Adolescents: enquête nationale. La Doc Française, Inserm.

[20] Choquet M, Lagadic C (1999). Evaluation en milieu scolaire d’un programme de prévention primaire en matière de toxicomanie. Obs Français Des Drog Des Toxicom.

[21] Norman GR, Sloan JA, Wyrwich KW (2003). Interpretation of changes in health-related quality of life: the remarkable universality of half a standard deviation. Med Care.

[22] Cohen J (1969). Statistical power analysis for the behavioural sciences.

[23] Wichstrøm L, Skoe E, von der Lippe A (1998). Self-concept development in adolescence : do American truths hold for Norwegians ?. Personality development in adolescence. A cross national and lifespan perspective.

[24] Harter S (1999). The construction of the self : a developmental perspective.

[25] Seidah A, Bouffard T, Vezeau C (2004). Perceptions de soi à l'adolescence : différences entre filles et garçons. Enfance.

[26] Frost J, McKelvie S (2004). Self-esteem and body satisfaction in male and female elementary schools, high school and university students. Sex Roles.

[27] Robins RW, Trzesniewski KH, Tracy JL (2002). Global self-esteem across the life span. Psychol Aging.

[28] Fourchard F, Courtinat-Camps A (2013). L’estime de soi globale et physique à l’adolescence. Neuropsychiatr Enfance Adolesc.

[29] Harter S, Delachaux N (1998). Comprendre l’estime de soi de l’enfant et de l’adolescent: considérations historiques, théoriques et méthodologiques. Estime soi Perspect. développementales.

[30] McVey GL, Davis R, Tweed S, Shaw BF (2004). Evaluation of a school-based program designed to improve body image satisfaction, global self-esteem, and eating attitudes and behaviors: a replication study. Int J Eat Disord.

[31] Niaraki FR, Rahimi H (2013). Effect of life skill training on self -esteem of high school students in Iran. Eur Online J Nat Soc Sci.

[32] Zangirolami F, Iemmi D, Vighi V, Pellai A (2018). On behalf of life skills group ASL Sondrio. Evaluating self-esteem modifications after a life skills-based education intervention. Minerva Pediatr.

[33] Bailly D, Choquet M, Roehrig C (2015). Evaluation du Programme espace (Education, Sensibilisation et Prévention Alcool au Collège avec l’appui de l’Environnement). Obs Régional La Santé Du Limousin.

[34] Dorard G, Bungener C, Corcos M, Berthoz S (2014). L'Encéphale.

[35] Masselink M, Van Roekel E, Oldehinkel AJ (2018). Self-esteem in early adolescence as predictor of depressive symptoms in late adolescence and early adulthood: the mediating role of motivational and social factors. J Youth Adolesc.

[36] Diener ED, Suh E (1997). Measuring quality of life: economic, social and subjective indicators. Soc Indic Res.

[37] Karatzias A, Chouliara Z, Power K, Swanson V (2006). Predicting general well-being from self-esteem and affectivity: an exploratory study with Scottish adolescents. Qual Life Res.

[38] Hankin BL, Abramson LY, Moffitt TE (1998). Development of depression from preadolescence to young adulthood: emerging gender differences in a 10-year longitudinal study. J Abnorm Psychol.

